# Supplementary material for: High-Density Patterned Array Bonding through Void-Free Divinyl Siloxane Bis-Benzocyclobutene Bonding Process
Source: Polymers (Basel). 2021 Oct 21;13(21):3633. doi: 10.3390/polym13213633 (PMC8588381; doi:10.3390/polym13213633)
Supplement: Supplementary file 1 [file polymers-13-03633-s001.zip › polymers-1405045-supplementary.pdf]

Supporting Information

High-Density Patterned Array Bonding Through Void-Free Divinyl Siloxane Bis-Benzocyclobutene Bonding Process

Nam Woon Kim[1], Hyeonjeong Choe[2], Muhammad Ali Shah[1], Duck-Gyu Lee[1,*] and Shin Hur[1,3,*]

[1] Department of Nature-Inspired System and Application, Korea Institute of Machinery and Materials, Daejeon 34103, Republic of Korea

[2] Department of Drug Discovery, Korea Research Institute of Chemical Technology, Daejeon 34114, Republic of Korea

[3] Department of Nano-Mechatronics, University of Science and Technology, Daejeon 34113, Republic of Korea

* Corresponding author. E-mail: educk9@kimm.re.kr, shur@kimm.re.kr

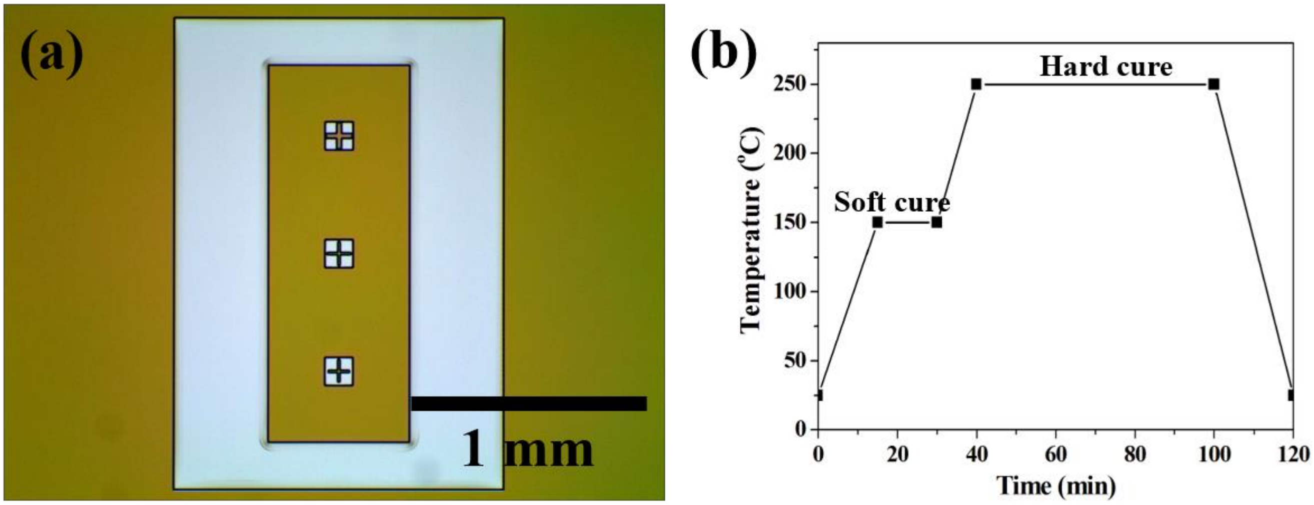

Figure S1. (a) DVS-BCB pattern alignment key and (b) temperature curing process for DVS-BCB bonding.

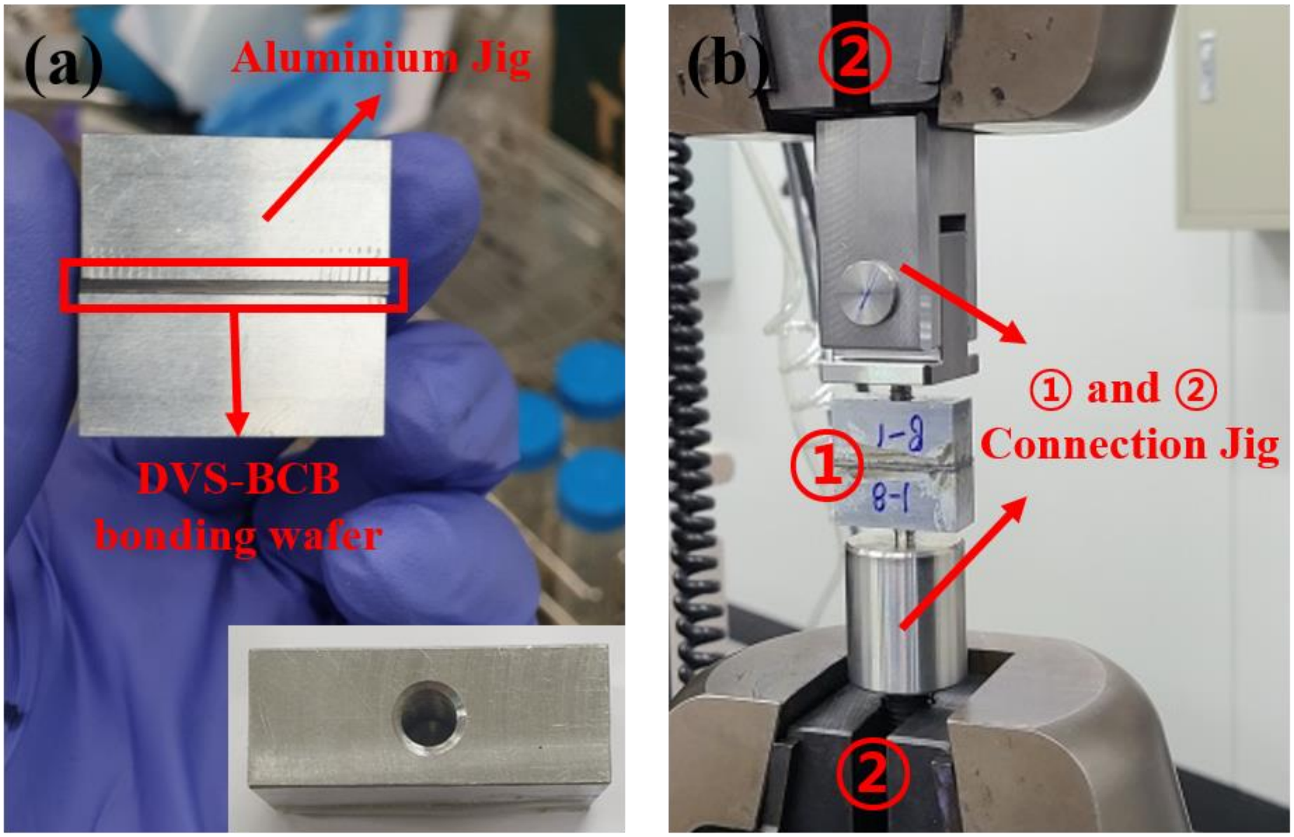

Figure S2. Experimental setup for the measurement of DVS-BCB bonding adhesion. (a) DVS-BCB-bonded wafer and aluminum jig connected using AXIA EP-04 epoxy. (b) DVS-BCB-bonding-wafer–aluminum-jig connected to universal testing machine using stainless steel connection jig.

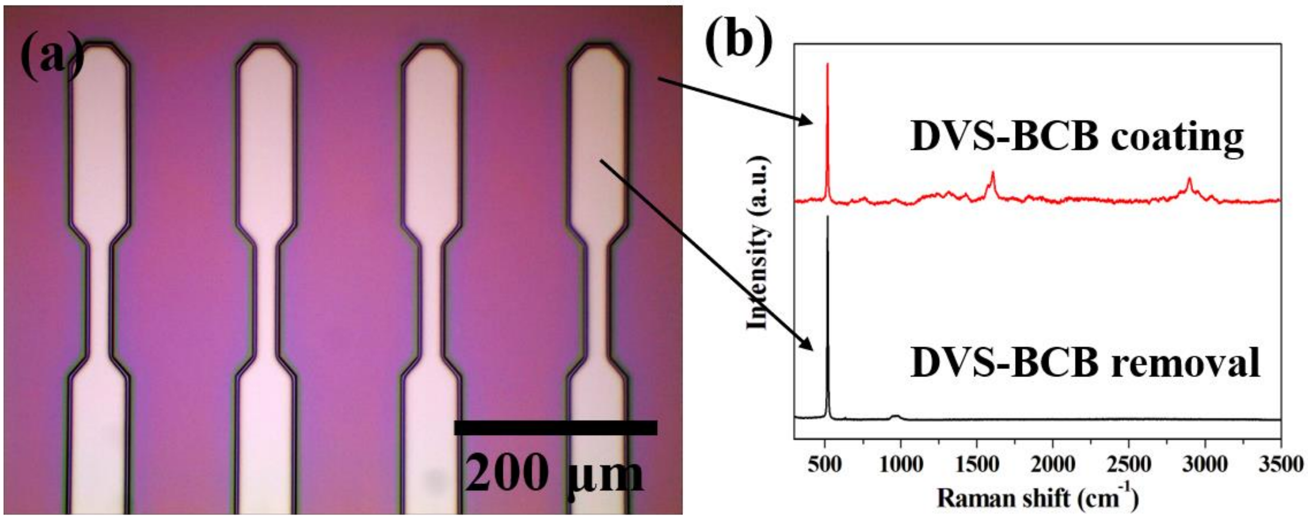

Figure S3. Raman spectra of the develop process results for the DVS-BCB patterns. (a) DVS-BCB pattern array microscopy image of the Raman measurement position. (b) Raman spectra with and without the DVS-BCB layer.

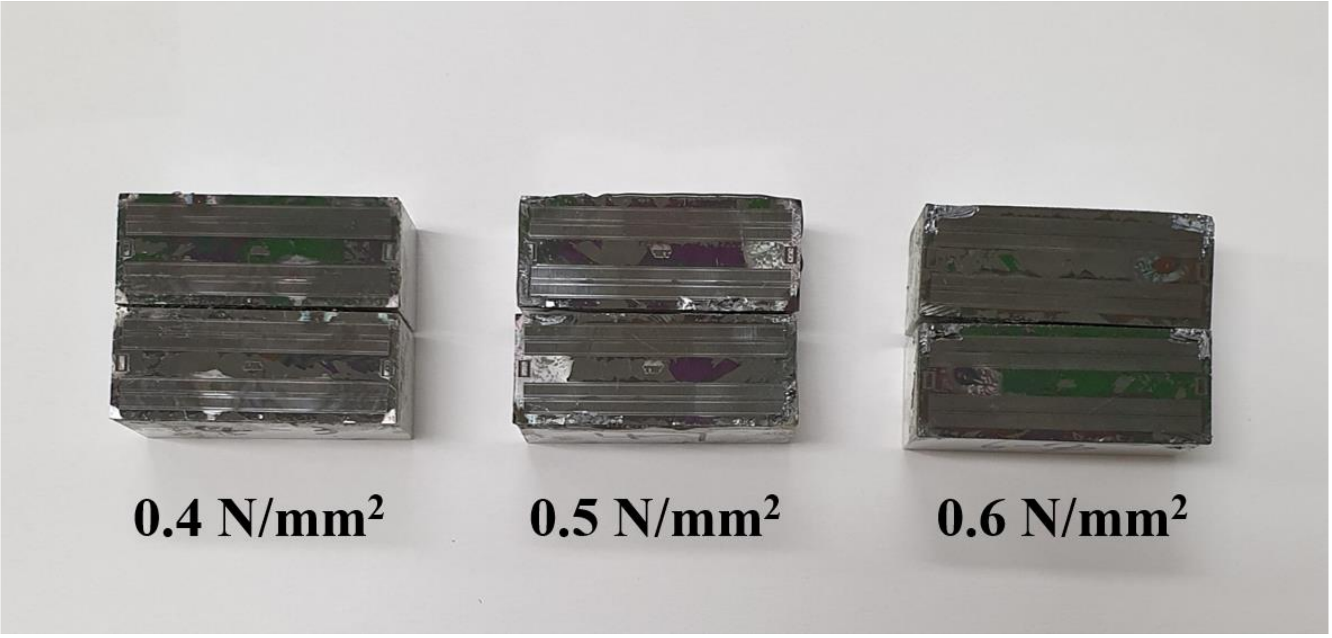

Figure S4. Optical images of samples detached after adhesion testing of DVS-BCB bonding.

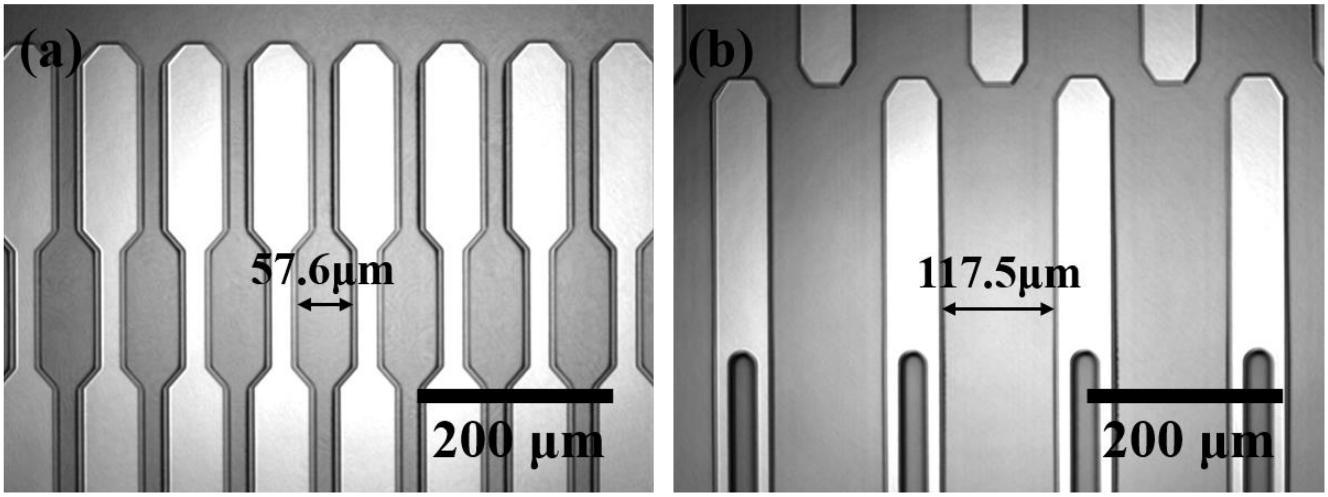

Figure S5. Near-infrared confocal laser microscopic images of the DVS-BCB bonding layer fabricated with a compressive pressure of 0.6 N/mm^2. (a) Super high-density patterns and (b) linear patterns.
